# Extended Complex Temporomandibular Joint Reconstructions Exploiting Virtual Surgical Planning, Navigation Assistance, and Custom-Made Prosthesis: A Comprehensive Protocol and Workflow

**DOI:** 10.3390/jpm13060931

**Published:** 2023-05-31

**Authors:** Luca Raccampo, Salvatore Sembronio, Alessandro Tel, Massimo Robiony

**Affiliations:** Maxillofacial Surgery Department, Academic Hospital of Udine, 33100 Udine, Italy

**Keywords:** TMJ prosthesis, eTMJR, virtual surgical planning, mandibular reconstruction, patient-specific surgery, computer-aided design

## Abstract

Background: Alloplastic temporomandibular joint (TMJ) replacement is a well-established procedure in maxillo-facial surgery. However, the surgical management of large excision in this area requires complex reconstruction beyond the standard TMJ prosthesis. Objective: This study aims to describe the design and the consequential application of a protocol which involves the use of computer-assisted surgery tools to best face complex TMJ reconstruction (TMJR). Preoperative accurate study of every single case and intraoperative check of the surgical act are nowadays essential to perform such delicate surgical procedures. Materials and Methods: The study is a retrospective and single institution case series. The various processes of the management and planning of extended TMJ reconstruction (eTMJR) are extensively described, from the preoperative clinical evaluation, imaging acquisition protocols and virtual surgical planning (VSP), focusing also on the intraoperative transfer of VSP using navigation and surgical guides. Results: We included nine patients with different pathologies which were candidates for eTMJR. Overall, the application of our protocol and workflow permitted the reduction of complications and pain, and the improvement of the maximum interincisal opening (MIO) of the patients, restoring patients’ masticatory function and esthetics. Conclusions: The eTMJR should be considered as a safe and reliable surgical management modality in selected patients with large temporomandibular joint and skull base (TMJ-SB) lesions. An accurate preoperative protocol and workflow is essential to perform such insidious and complex reconstruction. However, more extensive studies on this type of device have to be conducted in order to validate its real usefulness and indications.

## 1. Introduction

The temporomandibular joint (TMJ) is composed by the mandibular condyle which articulates with the glenoid fossa, a small and thin socket in the temporal bone of the skull base, along with the soft tissue component which provides static and dynamic stability including the disc, TMJ capsule with ligaments and muscle insertions. The glenoid fossa anatomically represents the boundary between the mandibular condyle and the middle cranial fossa. It is therefore evident how the lateral skull base forms part of the temporomandibular joint. Moreover, because of the close relationship between the condyle and middle cranial fossa, the term ‘‘temporomandibular joint and skull base’’ (TMJ-SB) has been proposed to define this region since 1974 [1]. This represents a key and surgically dangerous region; in particular, the area medial to the glenoid fossa is rich in large vessels and nerves, and while approaching the lateral skull base, the dura must always be kept intact [2] to avoid any communication with the intracranial space, which may represent the source of potentially life-threatening infections [3]. TMJ-SB can be affected by many clinical situations (articular ankylosis, neoplasms, congenital anomalies, traumas, osteomyelitis, etc.) which can impact on the esthetics of the area and, most importantly, masticatory function. Consequently, individuals with these pathologies often experience debilitating symptoms, including severe pain, swelling of the area, jaw clicking or popping, limited jaw movement, headaches, earaches, and even locked jaw. While non-surgical treatments are effective for many patients, they may prove insufficient in cases of advanced joint deterioration or significant structural abnormalities. TMJ replacement (TMJR) offers a comprehensive solution for individuals who have exhausted conservative options and still suffer from significant functional impairment and pain, or where there is the necessity to excise a lesion of the TMJ-SB area. Indeed, facing those pathologies, clinicians always have to consider the reconstruction of the excision area, with strategies taking into account the functional restoration of the TMJ. Historically, this was achieved performing an autologous replacement, while a shift to alloplastic TMJR has been observed in the last two decades, becoming a standard procedure for the treatment of TMJ disorders and the treatment of choice for end-stage TMJ diseases [4,5]. The autologous procedure is now almost entirely limited to pediatric patients or to those in which the alloplastic replacement has failed. However, standard TMJ prostheses may not fit the need to resolve an anatomical defect so large that it extends beyond the TMJ to the skull base, the lateral skull, and the mandible [6,7]. The introduction of virtual surgical planning (VSP) for TMJR has made it possible to fulfill any possible reconstructive needs [8]. This allows the design and manufacture of extended versions of custom-made TMJ prosthesis (eTMJR), which can satisfy the need of replacement of any section of the TMJ-SB besides the TMJ itself [9,10]. Specifically, eTMJR permits to replace not only articular components but also contiguous mandibular and/or temporal or zygoma bone defects with extended components for the ramus and fossa, respectively. This allows a functional and esthetically satisfying reconstruction for the patient. Moreover, the eTMJR has recently been the subject of various attempts to classify the type of prosthesis according to the defect to be fixed, which provides an indirect clue of the morphology of the reconstructive implant as shown [5,9,10]. Table 1 shows the first eTMJR classification system developed by Elledge et al. [9], which was later modified by Higginson et al. [10] (Table 2). We herein review our experience facing eTMJR, describing our protocol and workflow to perform reconstruction in this key and insidious area, highlighting and sharing pitfalls, and providing our tips regarding eTMJR.

## 2. Materials and Methods

### 2.1. Patient Population/Study Design

**Figure 1:**
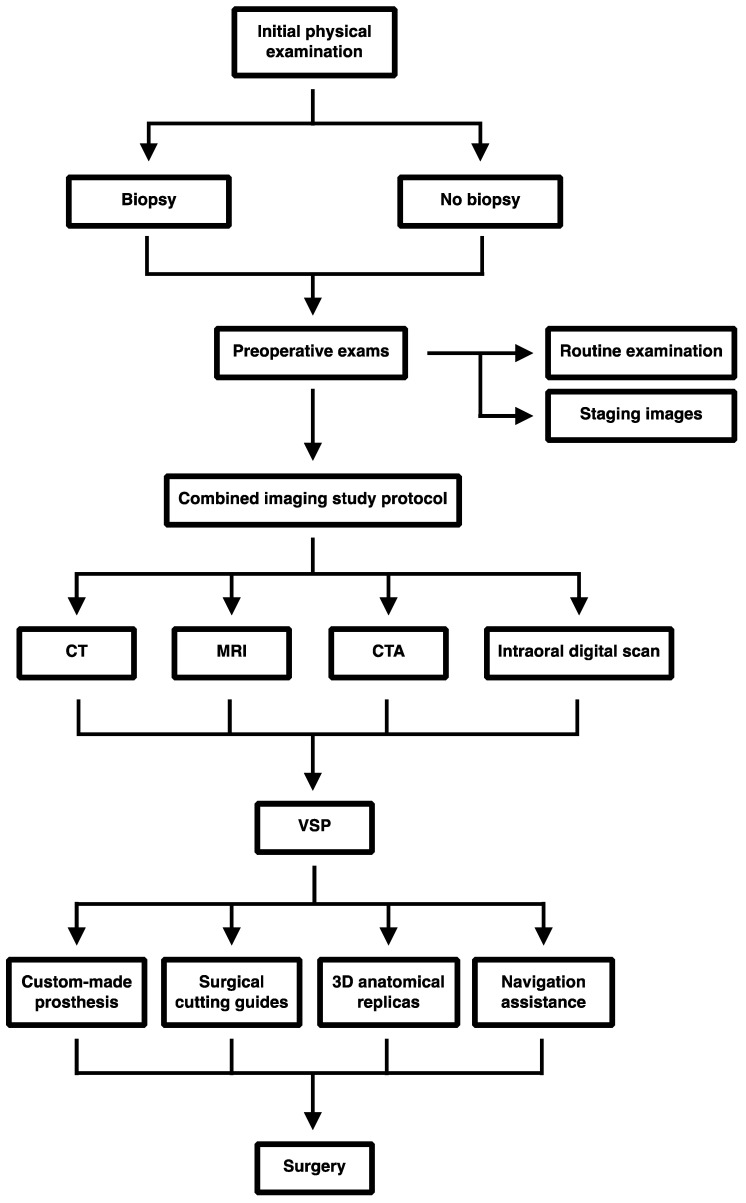
A schematic representation of our sequential workflow to manage eTMJR: Computer tomography (CT), magnetic resonance imaging (MRI), Computed Tomography Angiography (CTA), Virtual surgical planning (VSP).

This is a single institution, retrospective case series approved by the Institutional Review Board (IRB) of the University of Udine, conducted in the Department of Maxillo-facial surgery, Academic Hospital of Udine, from October 2017 to October 2022. Nine patients submitted to an eTMJR were selected. These patients were affected by various pathologies, benign and malignant, of the TMJ-SB area, defined superiorly and medially by the skull base, superiorly and laterally by the lateral skull, and inferiorly by the condyle, with contiguous potential extension to other parts of the jaw. Patients included in this case series had completed a follow-up period of at least six months, otherwise they would have been excluded from the study as well as patients with incongruous or missing clinical documentation. No other inclusion/exclusion criteria were established. The protocol and workflow is illustrated in detail below (Figure 1).

### 2.2. Physical Examination

A general and complete examination of the head and neck region was performed (e.g., search for oral lesions, swellings, malocclusion, laterocervical lymphadenomegalies, etc.). TMJ function was objectified clinically by measuring the mandibular range of motion, including maximum incisal opening (MIO) defined as the distance between the central incisors when the mouth is fully open, and lateral and protrusive movements were also assessed. Patients were asked to assess pre-auricular pain using a visual analogue scale (VAS), as well as masticatory function and diet. In cases of suspect malignancies, an incisional biopsy was performed, while in cases of benign pathologies such as ankylosis, this step was avoided.

### 2.3. Preoperative Exams

#### 2.3.1. Routine Examination

Each patient underwent routine preoperative examinations and assessments such as laboratory tests, echocardiograms, chest X-rays, anesthesiologic evaluation, general and rehabilitation evaluation, dietary evaluation, and in some cases, an electrophysiological study of the facial nerve.

#### 2.3.2. Staging Images

If the presence of malignancy was confirmed by biopsy examination, the patient underwent tumor-staging imaging examinations. A computer tomography (CT) scan of the neck, thorax, and abdomen, the most frequent sites of metastases of head and neck malignancies, were usually performed.

#### 2.3.3. Combined Imaging Study Protocol

In order to perform a correct and accurate VSP, the use of multiple imaging methods and specific acquisition protocols was necessary. Each anatomical structure of the affected region must be isolated and identified. This became particularly important in the subsequent VSP phase to permit precisely identifying the boundaries of the lesion and its relationship with adjacent structures. For this purpose, when planning an ETMJR, the patient normally underwent the following imaging examinations:

- CT: An ultrathin CT was performed, aiming to identify the skeletal structures with the following parameters: slice thickness = 0.625 mm, matrix = 512 × 512 px.
- Magnetic resonance imaging (MRI): A 1.5-Tesla system MRI (Aera; Siemens; Erlangen, Germany) was performed. Multiple sequences were acquired in order to best represent the different anatomical structures:
  ○ Soft tissues: After contrast medium administration, a 3D-VIBE T1-w sequence was acquired with a slice thickness of 1 mm and a matrix of 512 × 512 px.
  ○ Vascularization: Being a particularly delicate area considering the vascular and other structures present in the area, these must be identified. Medially to the glenoid fossa, besides the internal maxillary artery, there are the foramen ovalis, through which there is the mandibular nerve V3 (third division of the trigeminal nerve CN V), and the the foramen spinosum, which gives passage to the middle meningeal artery. Posteromedially instead, there are the foramen lacerum; the carotid canal, which contains the internal carotid artery; the jugular hole, which gives passage to the glossopharyngeal nerve (CN IX), vagus nerve (CN X), and accessory nerve (CN XI), and in its posterolateral originating from the sigmoid sinus, gives rise through the hole to the internal jugular vein; the stylomastoid hole, which contains the stylomastoid artery and the facial nerve (CN VII). So, to segment and reconstruct arterial vasculature, a 3D time-of-flight (TOF) MRI with the following parameters was acquired: TR = 25.0 ms; TE = 7.15 ms; slice thickness = 0.5 mm; in-plane resolution: 0.4 × 0.4 mm; slice GAP = −25%; matrix 256 × 256 px. To highlight venous structures, a phase-contrast MR venography sequence was performed.
- Computed Tomography Angiography (CTA): When a free bone flap was planned (e.g., free fibula flap, free iliac crest flap, etc.) in order to perform a mixed autologous/alloplastic eTMJR, the patient underwent a CTA, given the need to know the anatomy of the vascular pedicle.

#### 2.3.4. Intraoral Digital Scanning and Virtual Bite Registration

Dental anatomy reconstructed from a CT scan may originate suboptimal results due to a variety of reasons, such as limited image resolution, the creation of artefacts from metal dental restorations and orthodontic brackets, and the separation of upper and lower teeth in a closed bite position. For this reason, a digital acquirement of dental cusp anatomy using an intraoral scanner was performed (Carestream CS3600, Carestream Health Inc, Rochester, NY, USA). The resulting Standard Tessellation Language (STL) files were merged with the CT model and provide an increased detail of the dental cusps.

### 2.4. Virtual Surgical Planning

**Figure 2:**
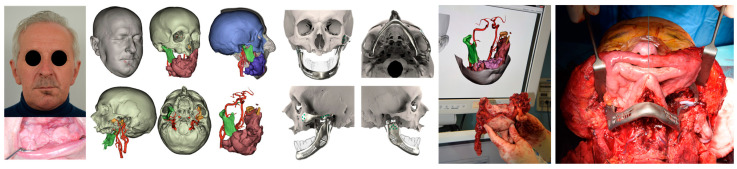
Patient 4 complete eTMJR workflow highlighting the very accurate VSP transferred into the operating room aided by intraoperative navigation and surgical cutting guides.

Recent progress in computer-aided technologies permitted to surgeons and engineers to integrate various imaging information in a single 3D planning following a multi-modality imaging model. CT and MR image output, as Digital Imaging and COmmunications in Medicine (DICOM) files, were then imported into Mimics (Materialise, Leuven, Belgium) and coregistered within the same coordinate system to achieve matched superimposition. First, a CT scan was segmented using a thresholding algorithm within the bone tissue Hounsfield unit (HU) range. As in standard TMJR, the resulting segmentation mask was then split into a mandible and skull subunit. In the same way, any bony mass or region of interest that we needed to excise was isolated. At this point, intraoral scanning is accurately aligned on the dental cusps retrieved from the CT scan using a point-to-point method, refined with an iterative closest point algorithm, and then merged with the CT scan. For pathologies involving the soft tissues, the masses were precisely segmented using semiautomatic methods based on AI (artificial intelligence)-powered smart brushes, while the MRI was already matched before. Vessels were virtually reconstructed using dynamic thresholding methods, applied to TOF sequences, exploiting detection methods based both on voxel contiguity and isointensity (Figure 2). Segmentation mask processing was thoroughly performed, especially to vessels in order to isolate relevant branches, and in the end, all the masks were carefully inspected for ideal correspondence with each set of DICOM images and then singularly exported as STL files. If a mixed autologous/alloplastic TMJR using a bony free flap was planned, image data were retrieved from the CTA. Then, the bone surfaces were reconstructed using a pre-contrast scan, while the vasculature and pedicle information were obtained by a contrast-enhanced scan in arterial phase.

### 2.5. Surgical Guides and eTMJR Prosthesis

**Figure 3:**
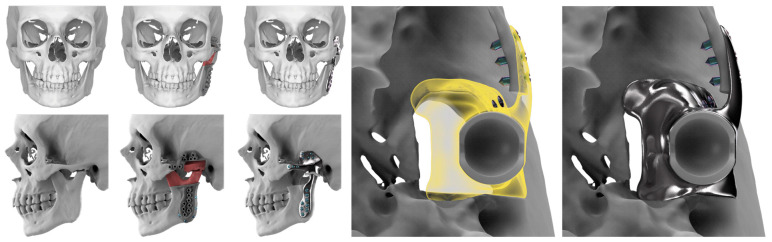
eTMJR accomplished in patient 9 which required a reconstruction of the skull base, zygomatic arch, and temporal bone.

STL files of the objects obtained by Mimics were then imported in 3-matic (Materialise, Leuven, Belgium). Simple resections were easily definable by simply positioning a cutting plane. While for complex osteotomies, we proceeded using a freehand brush poly-marking tool, drawing complex shapes, and defining curved resection profiles in order not to be restricted to simple planes. Each patient underwent an individualized approach simulation depending on the location of the disease. At this point, the project was transmitted on to an external company (Sintac-GPI, Trento, Italy) that produced the implant, and we proceeded to finalize the modeling of the custom-made prosthesis. Once the planning and the custom-made prosthesis design was approved by the surgeon, the implant was additively manufactured using metal 3D printing, and includes multiple components: the glenoid fossa component, which could extend medially to the skull base (Figure 3), but also superiorly to reconstruct defect of the zygoma and/or the temporal bone, was made of ultra-high-molecular-weight polyethylene (UHMWPE) material while its cranial extension was usually composed by titanium alloy (Ti-6Al-4V), and the condyle/ramus component, with its possible extended distal mandibular component, was usually composed by titanium alloy (Ti-6Al-4V). Fixation of these implants to the bone was achieved using screws of various size and length, and a hole was drilled in the condylar component of the prosthesis to facilitate the fixation of the prosthesis to the glenoid fossa component, thus avoiding dislocation of the implant. The characteristics of every eTMJR reported in this study are reported in Table 3.

### 2.6. D Printing

Using 3-matic (Materialise, Leuven, Belgium) custom-fitted surgical guides were designed to guide the planned osteotomies and drilling of screw holes, allowing to simultaneously secure the surgical guide and fix the prosthesis into the planned position. These guides were then printed using stereolithographic 3D printing (Formlabs, Somerville, MA, USA). Replicas of the various components, including the skull and the mandible with and without the resection, were also 3D printed and then sent for sterilization so that they were available in the operating theater. Once manufactured, the prosthesis, as well as the cutting guides and the replicas, were sent to our department. The entire process from the beginning of VSP to the delivery of the implants took approximately one week.

### 2.7. Navigation Assistance Setup

Navigational assistance is part of our workflow, permitting us to evaluate positional accuracy in relation to the VSP. Using Brainlab Elements (Brainlab, Munich, Germany), STLs from the virtual plan were imported into the navigation project being perfectly aligned with corresponding DICOM data. The entire navigation project was loaded in the navigation system.

### 2.8. Surgery

**Figure 4:**
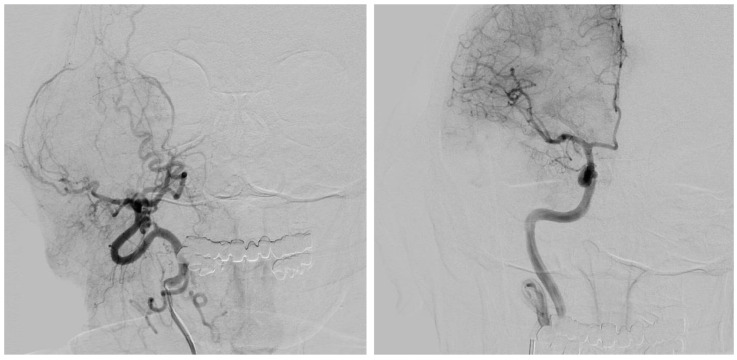
Selective embolization of right IMAX, STA, and MMA ostium in patient 1.

**Figure 5:**
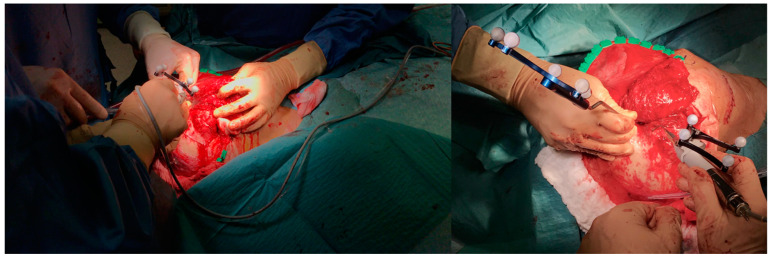
Navigated osteotomy of a bony mass of the TMJ-SB area using a piezoelectric instrument (Piezosurgery-Mectron Medical Technology, Genoa, Italy).

A further preoperative step consisting of selective embolization of the internal maxillary artery (IMAX), superficial temporary artery (STA), and middle meningeal artery ostium (MMA) was usually performed to minimize the risk of bleeding during eTMJR procedure (Figure 4). This operation was usually performed one or two days prior to prosthetic replacement surgery. On the day of the eTMJR, first, the intraoperative navigator (Brainlab, Munich, Germany) was installed with an optical stereoscopic camera mounted in the operating room, and a dynamic reference frame (DRF) was placed on the patient’s head. This allowed to track the probe independently from the patient’s head movements required in different moments by the surgeon. Calibration was performed using a surface-based registration method further improved with anatomical landmark recognition. All phases of the surgery were navigated according to the VSP. All patients received general anesthesia with naso-endotracheal intubation, or oro-endotracheal or percutaneous tracheostomy. The surgical approach varied depending on the location of the pathological process. Normally, a preauricular incision with a little temporal extension and retro/submandibular approach were performed to expose the TMJ and the ramus permitting to complete a standard TMJR. For eTMJR, on the other hand, the choice of surgical approach depends on several factors including the extension of the pathology, the bone and soft structures involved, and the reconstructive needs. If it was necessary to reconstruct the zygomatic arch or part of the temporal bone, the retro/submandibular access was often combined with a hemicoronal approach. Whereas, if the defect to be reconstructed extended beyond the ramus with different extension, the retro/submandibular approach was not sufficient and a classic cervicotomic access was necessary, often accompanied by a concomitant intraoral access. If the defect to be restored was complete or almost complete, it may be necessary to resort to a complete visor flap. Once the surfaces to be osteotomized were exposed, the cutting guides were positioned and fixed according to drilling guides and holes using titanium alloy screws that will then also be used to fix the prosthesis. Nerve stimulators or facial nerve monitors were used to mitigate the risk of facial nerve injury. Before the osteotomy, an intraoperative check of the osteotomies to be performed was carried out using navigation assistance. The osteotomy itself could be navigated using a piezoelectric instrument (Piezosurgery-Mectron Medical Technology, Genoa, IT) (Figure 5). Once the osteotomies had been performed, the prostheses were positioned and fixed using titanium alloy screws of various size and length according to the holes previously made thanks to the cutting guides. When an eTMJR was complemented by a free bone flap (free fibula flap, free iliac crest flap, etc.), specific cutting guides were designed and 3D printed. If it was planned to place implants in the free bone flap according to a pre-planned virtual wax, after discussion with the prosthodontist and integration into the VSP flow, this was performed in the same operation, once the flap had been harvested and mounted on the custom-made prosthesis. The prosthetic condyle was fixed with a single non-resorbable suture to the fossa component to prevent luxation of the alloplastic joint and postoperative sagging. Sometimes, we put a fat graft around the new joint to facilitate gliding and reduce the risk of bone heterotopic formation in case of a previously severe ankylotic joint. At the end of the procedure, an occlusion check was performed, and the surgical accesses were sutured. Immediately after the surgery, a CT scan was carried out and the prosthesis position was finally checked.

### 2.9. Follow-Up and Outcome Evaluation

Patients were subjected to a progressively less strict clinical follow-up for the first 6 months after surgery, weekly in the first month, and monthly thereafter, mainly assessing the possible occurrence of complications, quality of life, pain level through a visual analogue scale (VAS), masticatory function, and mandibular range of motion by measuring the MIO. A second CT scan was performed 6 months after surgery in order to verify the stability of the eTMJR. Patients usually returned to a normal diet within 1 month after surgery. If the clinical and radiological control at 6 months was satisfactory, the patient will be clinically evaluated every year with an annual follow-up CT scan for at least 5 years from surgery.

## 3. Results

Between October 2017 and October 2022, nine patients with different diseases involving the TMJ-SB were treated with an eTMJR implant, whose data are shown in Table 4. Once identified, patient records including operative reports, clinical charts, and imaging were examined to obtain the data. Patient age ranged from 24 to 71 years, with a median of 51.1 years. All patients suffered from different conditions affecting the TMJ-SB: ossifying fibroma, osteoma, sarcomatoid carcinoma, mucoepidermoid carcinoma, ankylosis, keratocysts, squamous cell carcinoma, fibrous dysplasia, and osteoblastoma. In total, 10 eTMJR were performed, 8 unilateral, and 1 bilateral following our protocol and workflow, and so all the computer-aided surgery tools were used in all cases according to the sequence detailed in the Materials and Methods section. Partial irreversible facial nerve palsy occurred in two out of nine patients, mostly due to the local extension of the disease, while only one patient lost their implant because of a retarded free flap loss and the consequential necrosis and infection which overlapped the prosthesis. No other implant failure or screw loosening of the fossa or mandibular components were observed. The eTMJR were classified using the two-component modified eTMJR classification proposed by Higginson et al. (Table 2) [10]. Three subjects were classified as F0-M3 eTMJR, two FT-M0, two FA-M0, one FA-M3, and one F0-M2. 

**Figure 6:**
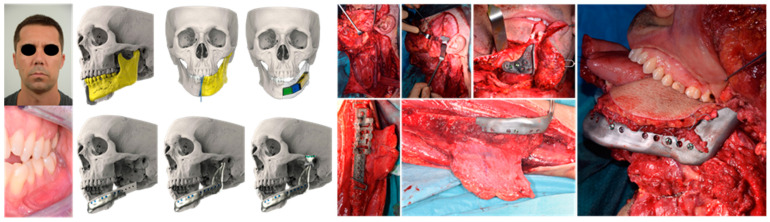
eTMJR accomplished in patient 9, which required a reconstruction of the skull base, zygomatic arch, and temporal bone. Autologous/alloplastic eTMJR of patient 3, for whom we designed a particular two-interlocking mandible component of the prosthesis which also had a dedicated internal flange to accommodate the fibula free flap properly harvested and cut, thanks to a specific cutting guide.

Two patients were also submitted to an hybrid autologous/alloplastic eTMJR using a fibula free flap (Figure 6). The MIO was assessed both preoperatively and postoperatively, showing a mean MIO preoperative of 23.5 mm, compared to 32.4 mm at follow-up. The patients reported an encouraging improvement in pain, decreasing significantly from a preoperative VAS mean score of 5.3 to a six-month follow-up of 1.8. Two patients had already completed the five-year follow-up period, while the patient with the shortest follow-up time was eight months. One patient died one year after the procedure due to the advancement of the disease. All the patients showed stable postoperative occlusion and normal hinge movement of the TMJ. 

## 4. Discussion

**Figure 7:**
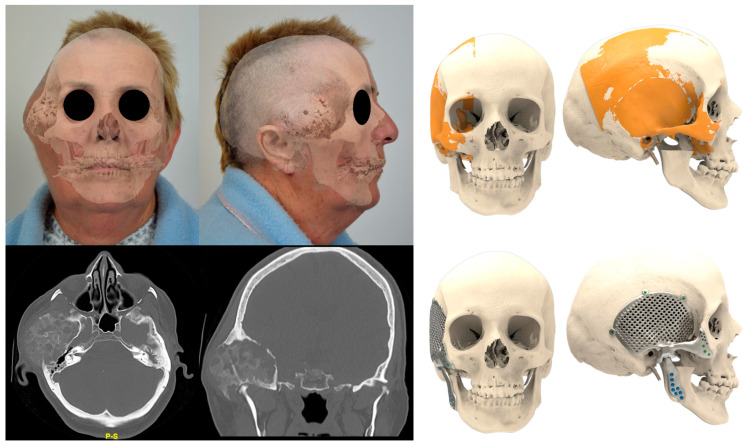
Patient 1 eTMJR planned by mirroring the contralateral side of the lateral skull to produce a satisfying functional and esthetic result.

TMJR surgery represents a challenge for every maxillo-facial surgeon, especially in cases where the TMJ anatomy is pathologically altered involving the whole TMJ-SB area, and thus performing a standard TMJR is nearly impossible. Particularly at the skull base, where vital structures lie in direct contiguity with the TMJ, more control and monitoring during drilling and cutting might increase patient safety and precise reconstruction. Moreover, patients that undergo TMJR are susceptible to potentially life-threatening vascular injury, most commonly of the IMAX. It is well established that preoperative embolization can reduce the risk of blood loss from inadvertent vascular injury and from surgical approach/dissection [11]. Cillo et al. reported that in addition to IMAX, the MMA also may be injured during TMJR which involves extensive dissection, as in an eTMJR case [12]. While the use of standard TMJR prosthesis is becoming an increasingly popular and used procedure, and its clinical indications for implantation are well established, a consensus on the use of eTMJR prostheses has yet to be developed [9]. It is also true that pathological conditions treatable with eTMJR are hugely different and more complex than those which require a standard TMJR. Such pathologies whose resection and concomitant reconstruction always have pitfalls and contingencies can occur in the operating room, even if an accurate VSP has been performed. Our proposed workflow aims to present the benefits and efficacy of a comprehensive eTMJR protocol to correct large defects involving the TMJ and adjacent structures. Intraoperative navigation, VSP, and 3D-printed surgical cutting guides and prostheses are well known to facilitate and increase accuracy in surgery, particularly in anatomical variances such as congenital deformities, ankylosis, or multiply operated joints [3]. Real-time navigation, in particular, is reported to greatly increase accuracy and precision in TMJR [13,14]. Although the application of our protocol allowed us to minimize obstacles and unexpected hitches in the operating room, intraoperative adjustments were necessary in some cases. It must also be said that these unforeseen events allowed us to re-evaluate and refine this protocol, thus implementing certain corrections or features in the prosthesis, VSP, cutting guides, etc. Adjustments, albeit minimal, on the glenoid fossa and mandibular component were the most frequently observed. We also experienced lack of manual control over the condyle position into the fossa component before fixation, and unpredictable reattachment of the elevator muscles of the jaw. We fixed these issues providing dedicated holes on the condylar head and in the gonial region which permits suture suspension of the mandibular component to the prosthetic fossa and proper fixation of the pterygomasseteric sling, as we performed in case 4, 6, and 8. The eTMJR also allows facial remodeling in cases where facial esthetic is particularly distorted, as in case 1 and 8. In these patients, the VSP mirroring tool and the possibility of retrieving jaw and skull models from an in-house software library to plan the best-matching eTMJR were crucial (Figure 7). Sanovich et al. has already reported improvements in maximal interincisal opening (MIO), pain recovery, diet restriction, and quality of life (QOL) in medium-term to long-term follow-up of TMJR patients [15]. Results of our cases demonstrate that MIO and pain levels were significantly improved, and an overall enhancement in the functional and esthetic variables of the patients was evident with the implantation of the eTMJR prostheses. Furthermore, in contrast to autologous reconstruction, eTMJR allows early mobilization, which has been shown to improve functional outcomes [16,17], as we have also observed in our patients. Elledge et al. first developed a useful eTMJR classification system (Table 1) stating that any classification system for eTMJR has to be “unambiguous and easy to use; exhaustive and mutually exclusive so that each possibility exists in only one class; clinically relevant and appropriate; and flexible enough to accommodate any advances or changes in technology” [9]. This first classification attempt was composed by two components: the “M” component for the mandibular component of the prosthesis, and the “F” for the fossa component. Each of these two parameters is further divided into four sub-categories in order of the size of the defect to be filled. This classification has as an objective to delineate the different levels of resection of the fossa, skull base, and mandible. Later, the F parameter of this classification system was modified by Higginson et al. [10] in a three-tiered mode attempting to simplify the fossa classification and facilitate communication between clinicians and manufacturers (Table 2). Another subclassification system of eTMJR was proposed by Mommaerts et al. [5], which considered three potential obstacles: contour corrections, occlusal adjustments, and simultaneous contralateral mandibular osteotomy. In our opinion, there is a further point of reflection that might be useful to implement this classification. In fact, all these classifications do not take into account the possibility of hybrid, autologous, and alloplastic eTMJR, as, for example, we performed in the cases of patients 3 and 8 [18]. In these cases, the eTMJR mandibular body has been designed and shaped to support a free fibula flap, thus virtually recreating the alveolar bone process, permitting also contextual or delayed dental rehabilitation. Few authors reported that satisfactory rehabilitation can be achieved through this hybrid technique [4,13]. With the increasing popularity of this type of procedure, this aspect should be, in our opinion, taken into account within the eTMJR classification.

## 5. Conclusions

The eTMJR should be considered as a safe and reliable management modality in selected patients with large TMJ-SB area defects. In our experience, patients undergoing eTMJR had an improvement of the MIO and in pain outcome, and consequently in their quality of life. An accurate preoperative protocol and workflow is essential to perform such insidious and complex reconstruction. However, more extensive studies on this type of device have to be conducted, in order to validate its real usefulness and indications. As medical advancements continue to evolve, the field of eTMJR holds the potential to offer even more precise and effective interventions which should enable patients to regain optimal jaw function and live pain-free lives.

## Figures and Tables

**Table 1:** First proposed eTMJR classification by Elledge et al. in 2018.

Category	Description
Fossa component:	
F0	Standard fossa component (contained within fossa)
FA	Extended fossa component extending into the site of the zygomatic arch
FT	Extended fossa component extending to cover a defect in the temporal bone
Mandible (ramus) component:	
M0	Standard condyle-ramus component (proximal to angle of mandible)
M1	Extended proximal to ipsilateral mental foramen
M2	Extended proximal to contralateral mental foramen
M3	Extended beyond contralateral mental foramen
M4	Total alloplastic mandible, including both condyles

**Table 2:** Modified 2021 eTMJR classification proposed by Higginson et al.

Category	Description
Fossa component:	
F0	Standard fossa component (contained within fossa)
F1	Extending anteriorly to but not beyond the articular eminence
F2	Extending beyond the articular eminence anteriorly (zygomatic arch defect)
F3	Temporal bone defect not including auditory apparatus +/− arch defect
F4	Temporal bone defect involving auditory apparatus +/− arch defect
F5	Temporal defect extending to jugular foramen
Mandible (ramus) component:	
M0	Standard condyle-ramus component (proximal to angle of mandible)
M1	Extended proximal to ipsilateral mental nerve foramen/region
M2	Extended proximal to contralateral mental nerve foramen/region
M3	Extensive extending beyond contralateral mental nerve foramen/region
M4	Total alloplastic mandible (including both condyles)

**Table 3:** Custom-made eTMJR individual characteristics. The * stands for Autologous/alloplastic eTMJR.

ID	Age	eTMJR Class	Image	Reconstructed Structures	Fossa Materials	Condyle Materials	Screws and Fixation Holes
1	71	FT-M0	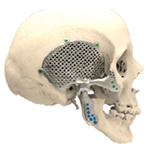	Temporal bone, aygomatic arch, glenoid fossa and condyle	Unalloyed titanium mesh temporal and zygomatic backing capping the UHMWPE fossa	Cobalt-chromiun-molybdenum alloy condylar head and Titanium alloy body	Skull component: 62.0 mm titanium screws. Manibular component: 82.7 mm titanium screws
2	58	FA-M0	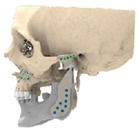	Glenoid fossa with medial extension and condyle	Alloyed titanium backing capping the UHMWPE fossa	Cobalt-chromiun-molybdenum alloy condylar head and Titanium alloy body	Skull component: 52.0 mm titanium screws. Manibular component: 82.7 mm titanium screws
3	41	FO-M3 *	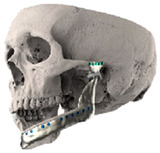	Glenoid fossa and complete left hemimandible	Alloyed titanium backing capping the UHMWPE fossa	Cobalt-chromiun-molybdenum alloy condylar head and Titanium alloy body consisting of two interloching pieces	Skull component: 52.0 mm titanium screws. Manibular component: 112.7 mm titanium screws
4	71	FA-M3	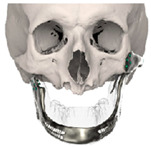	Left zygomatic arch, glenoid fossa and complete left hemimandible extended to the right hemimandible ramus	Alloyed titanium zygomatic backing capping the UHMWPE fossa	Titantium alloy	Skull component: 82.3 mm titanium screws. Manibular component: 62.7 mm titanium screws
5	26	FA-M0	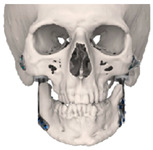	Temporal bone, zygomatic arch, glenoid fossa and condyle bilatreally	Alloyed titanium zygomatic backing capping the UHMWPE fossa	Cobalt-chromiun-molybdenum alloy condylar head and Titanium alloy body	Skull component: 172.0 mm titanium screws. Manibular component: 192.7 mm titanium screws
6	54	F0-M2	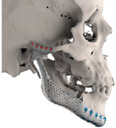	Glenoid fossa and complete right hemimandible	Alloyed titanium backing capping the UHMWPE fossa	Cobalt-chromiun-molybdenum alloy condylar head and Titanium alloy mesh body	Skull component: 52.3 mm titanium screws. Manibular component: 92.7 mm titanium screws
7	63	F0-M3	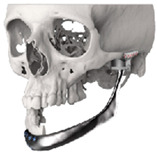	Glenoid fossa and complete left hemimandible	Alloyed titanium backing capping the UHMWPE fossa	Cobalt-chromiun-molybdenum alloy	Skull component: 52.3 mm titanium screws. Manibular component: 82.7 mm titanium screws
8	24	F0-M3 *	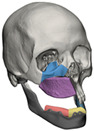	Glenoid fossa and complete right hemimandible extended to the left hemimandible ramus	Cobalt-chromium-molybdenum alloy backing capping the UHMWPE fossa	Cobalt-chromiun-molybdenum alloy	Skull component: 52.3 mm titanium screws. Manibular component: 112.7 mm titanium screws
9	52	FT-M0	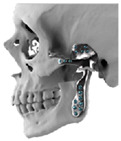	Temporal bone, zygomatic arch, glenoid fossa extended medially and condyle	Cobalt-chromium-molybdenum alloy backing capping the UHMWPE fossa	Cobalt-chromiun-molybdenum alloy	Skull component: 82.3 mm titanium screws. Manibular component: 82.7 mm titanium screws

**Table 4:** Characteristics of patients focusing on disease, eTMJR class, MIO, and pain postoperative changes; the * on the eTMJR class indicates that a hybrid, autologous, and alloplastic eTMJR was performed.

ID	Age	Gender	Diagnosis	Complications	eTMJR Class	MIO before Surgery	MIO after Surgery	VAS Preoperatory	VAS at 6 Months	Follow-Up
1	71	Female	Ossifying fibroma o right temporal bone	None	FT-M0	29 mm	35 mm	2	0	5 years
2	58	Male	Left condylar osteoma	Partial left facial palsy	FA-M0	20 mm	32 mm	7	2	5 years
3	41	Male	Sarcomatoid carcinoma of lef hemimandible	None	F0-M3 *	35 mm	35 mm	4	3	1 year
4	71	Male	High grade Mucoepidermoid carcinoma of the oral pelvis	Periprocedural pulmonary infection, Partial left facial palsy	FA-M3	27 mm	30 mm	7	5	4 years
5	26	Male	Bilateral severe TMJ ankylosis	None	FA-M0	8 mm	24 mm	8	1	4 years
6	54	Female	Left mandibular angle odontogenic keratocyst	None	F0-M2	36 mm	38 mm	5	3	3 years
7	63	Female	Infiltrating squamous cell carcinoma of the left cheek mucosa	Loss of the implant	F0-M3	23 mm	34 mm	5	2	2 years
8	24	Male	Right hemifacial fibrous dysplasia	None	F0-M3 *	24 mm	31 mm	3	0	1 year
9	52	Female	Left articular eminence osteoblastoma	None	FT-M0	10 mm	33 mm	7	1	8 months

## Data Availability

Data available on request due to restrictions eg privacy or ethical. The data presented in this study are available on request from the corresponding author.
